# Impact of Mutagens on DNA Replication in Barley Chromosomes

**DOI:** 10.3390/ijms19041070

**Published:** 2018-04-03

**Authors:** Jolanta Kwasniewska, Karolina Zubrzycka, Arita Kus

**Affiliations:** Department of Plant Anatomy and Cytology, University of Silesia, Jagiellonska 28, 40-032 Katowice, Poland; karolinazubrzycka92@gmail.com (K.Z.); arkus@us.edu.pl (A.K.)

**Keywords:** barley, chromosome, DNA replication pattern, EdU, mutagens

## Abstract

Replication errors that are caused by mutagens are critical for living cells. The aim of the study was to analyze the distribution of a DNA replication pattern on chromosomes of the *H. vulgare* ‘Start’ variety using pulse 5-ethynyl-2′-deoxyuridine (EdU) labeling, as well as its relationship to the DNA damage that is induced by mutagenic treatment with maleic hydrazide (MH) and γ ray. To the best of our knowledge, this is the first example of a study of the effects of mutagens on the DNA replication pattern in chromosomes, as well as the first to use EdU labeling for these purposes. The duration of the cell cycle of the *Hordeum vulgare* ‘Start’ variety was estimated for the first time, as well as the influence of MH and γ ray on it. The distribution of the signals of DNA replication along the chromosomes revealed relationships between DNA replication, the chromatin structure, and DNA damage. MH has a stronger impact on replication than γ ray. Application of EdU seems to be promising for precise analyses of cell cycle disturbances in the future, especially in plant species with small genomes.

## 1. Introduction

Data regarding the effects of mutagens on plant nuclear genomes and DNA replication are of great importance. The spatiotemporal patterns of DNA replication in nuclei were recently characterized in detail in control cells [1], as well as in relation to DNA damage and mutagenesis [2] using a quantitative analysis. However, to date there is no similar data on the effects of mutagens on the pattern of DNA replication on chromosomes. Analyses of the distribution of the signals of DNA replication on the chromosomes can be more informative when exploring the relationships between DNA replication, the chromatin structure, and DNA damage than studies using non-dividing cells.

Until now, the localisation of replicated chromatin was only possible using bromodeoxyuridine (BrdU). One of the disadvantages of using BrdU is degradation of the chromatin structure during denaturation step, which is especially inconvenient in the context of an analysis of DNA damage during mutagenesis. The relatively large size of the detection sites caused by the need to use specific antibodies to detect BrdU is an unfavorable feature of an analysis of DNA replication sites, especially in the case of an analysis of the signals in chromosomes. Currently, the “click” reaction using 5-ethynyl-2′-deoxyuridine (EdU) [3,4] is commonly used. Its good preservation of chromatin and high resolution make this technique useful in a detailed analysis of the effects of mutagens on the S-phase [2].

In this study, we present the distribution of the DNA replication pattern on chromosomes using pulse EdU labeling and analyze its relationship with the DNA damage that is induced by mutagenic treatment with maleic hydrazide (MH) and γ ray. To the best of our knowledge, this is the first example of a study of the effects of mutagens on the DNA replication pattern in chromosomes, as well as the first to use EdU labeling for these purposes.

We used barley (*Hordeum vulgare* ‘Start’ variety, 2*n* = 14) as the model plant species. Barley, which is characterized by relatively large chromosomes and a specific heterochromatin distribution [5,6], is a convenient species for an analysis of the distribution of the DNA replication pattern along the chromosomes, as well as in the context of mutagenesis. Barley is regarded to be a model species in analyses of the cytogenetic effects of mutagens, especially due to its chromosome size. Chromosome rearrangements [7,8], as well as disturbances of the cell cycle [2] after mutagenic treatment, have previously been shown in barley cells. In our study, the duration of the cell cycle of the *Hordeum vulgare* ‘Start’ variety was estimated, as well as the influence of MH and γ ray on it, by applying the EdU method.

## 2. Results

**Figure 1:**
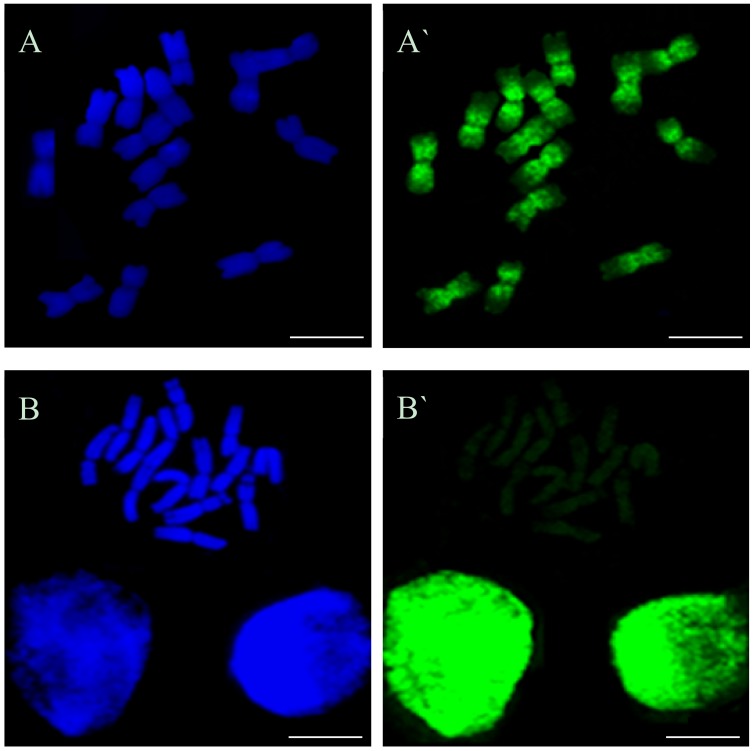
Barley metaphase cells from the control (untreated) roots with green Alexa Fluor 488 replication signals (**A**,**A`**) and with no signals (**B**,**B`**). (**A**,**B**) DAPI staining, all chromosomes stained. (**A`**,**B`**) results of 5-ethynyl-2′-deoxyuridine (EdU) incorporation and detection with Alexa Fluor 488 azide. Bars represent 10 μm.

The cells that passed through the S-phase during EdU incorporation were characterized by the presence of green Alexa Fluor 488 replication signals on the chromosomes (Figure 1A,A`). Cells with no replication signals on the chromosomes were also observed (Figure 1B,B`), which indicated that they were not in the S-phase during EdU incorporation.

### 2.1. The Frequency of Metaphases with Replication Signals

**Figure 2:**
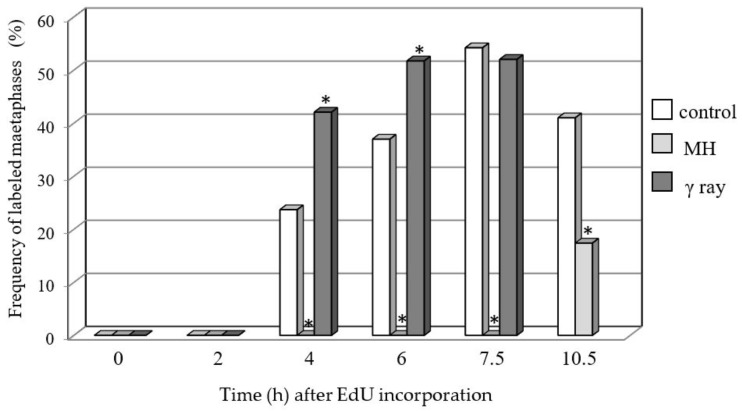
Frequencies of barley-labeled metaphase cells showing replication signals in the control, MH-, and γ ray-treated roots. Treated groups significantly different (*p* < 0.05) from the control are indicated by *.

The frequencies of the labeled metaphases in the control and MH- or γ ray-treated roots were analyzed at 0, 2, 4, 6, and 7.5 h after EdU incorporation (Figure 2). No metaphases with replication signals were observed at 0 and 2 h for the control or any of the mutagenic experimental groups. The first labeled metaphases in the control and γ ray-treated roots were observed at 4 h—their frequency was 23.7% in the control and 42.1% in the γ ray-treated cells. Similarly, at 6 h and 7.5 h, labeled chromosomes were observed only in the control and γ ray-treated roots. The frequency of the metaphases with replication signals in the control was significantly lower than in the γ ray-treated roots, both at 4 h and 6 h. At 7.5 h, the frequency of the labeled metaphases in the γ ray-treated cells was only slightly lower than in the control. Because no labeled metaphases were observed in the MH-treated roots even at 7.5 h, additional examination time points—9 and 10.5 h—were added. The first metaphases with labeled chromosomes after the MH treatment were observed at 10.5 h, and their frequency was 2.4 times lower than in the control roots at this time point.

### 2.2. Replication Pattern in Individual Chromosomes

**Figure 3:**
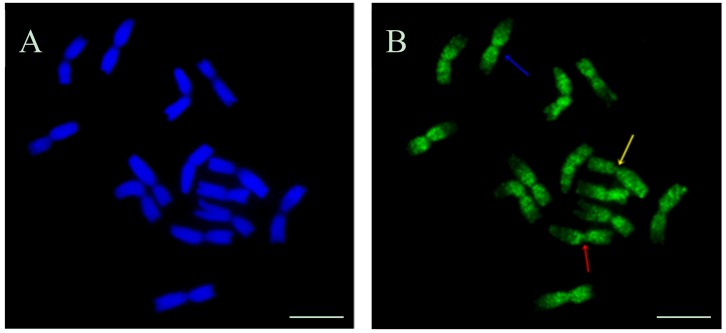
Different replication patterns of the barley chromosome within one metaphase cell. Replication signals were observed in the proximal and interstitial regions of the chromosome (blue arrow), in one entire chromosome arm, and in the proximal region of the second arm (red arrow) and in whole chromosomes (yellow arrow). (**A**) DAPI staining. (**B**) results of 5-ethynyl-2′-deoxyuridine (EdU) incorporation and detection with Alexa Fluor 488 azide. Bars represent 10 μm.

Different replication patterns were observed in the chromosomes in the barley roots within individual metaphases. An example of a metaphase cell with individual chromosomes that are is characterized by a few replication patterns is presented in Figure 3. Replication signals were observed in the proximal and interstitial regions of characterized chromosome (blue arrow), in one entire chromosome arm, and in the proximal region of the second arm (red arrow), as well as in whole chromosomes (yellow arrow).

### 2.3. Chromosome Replication Pattern in the Control and Mutagen-Treated Roots

**Figure 4:**
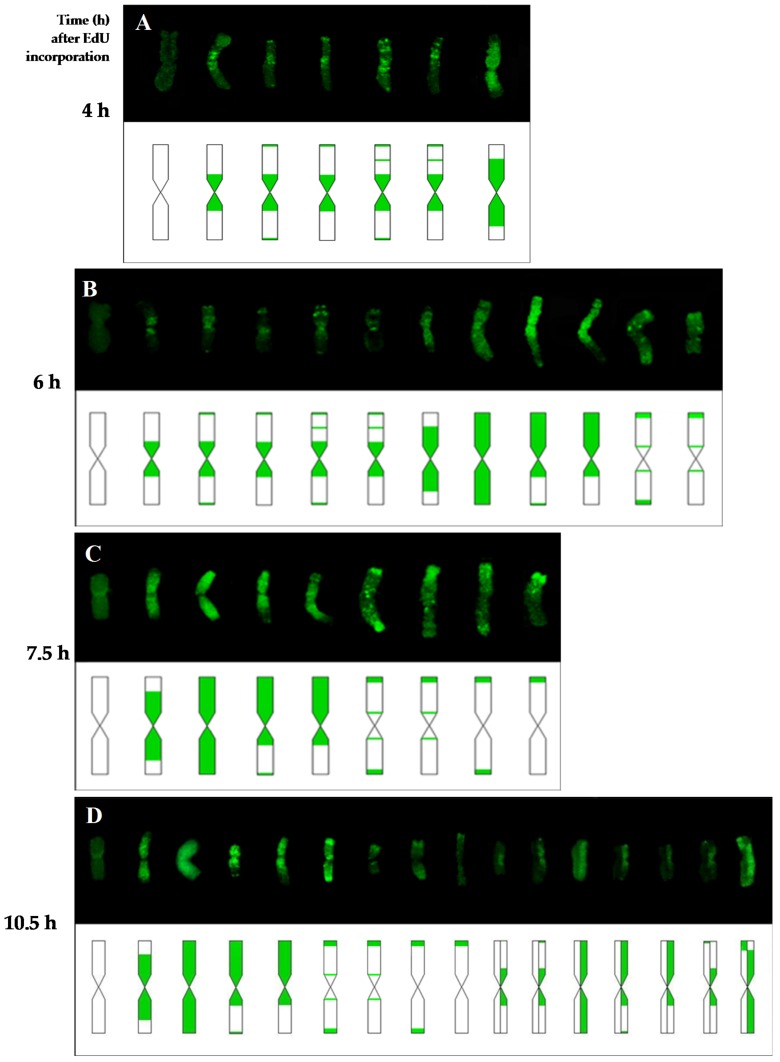
Types of replication patterns in barley chromosomes in the control roots at 4 h (**A**), 6 h (**B**), 7.5 h (**C**), and 10.5 h (**D**) after EdU incorporation. Chromosomes with different replication patterns and their corresponding schematic presentation are shown. Only one morphological type of chromosome is presented in order to simplify the scheme.

**Figure 5:**
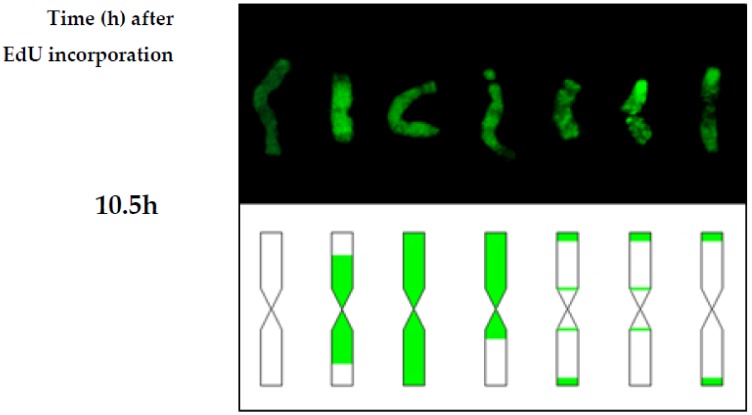
Types of replication patterns in the barley chromosomes in MH-treated roots at 10.5 h after EdU incorporation. Chromosomes with different replication patterns and their corresponding schematic presentation are shown. Only one morphological type of chromosome is presented in order to simplify the scheme.

**Figure 6:**
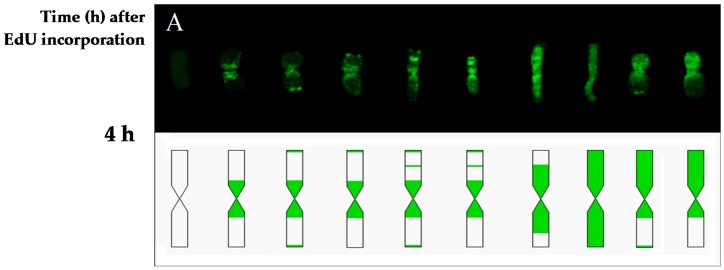

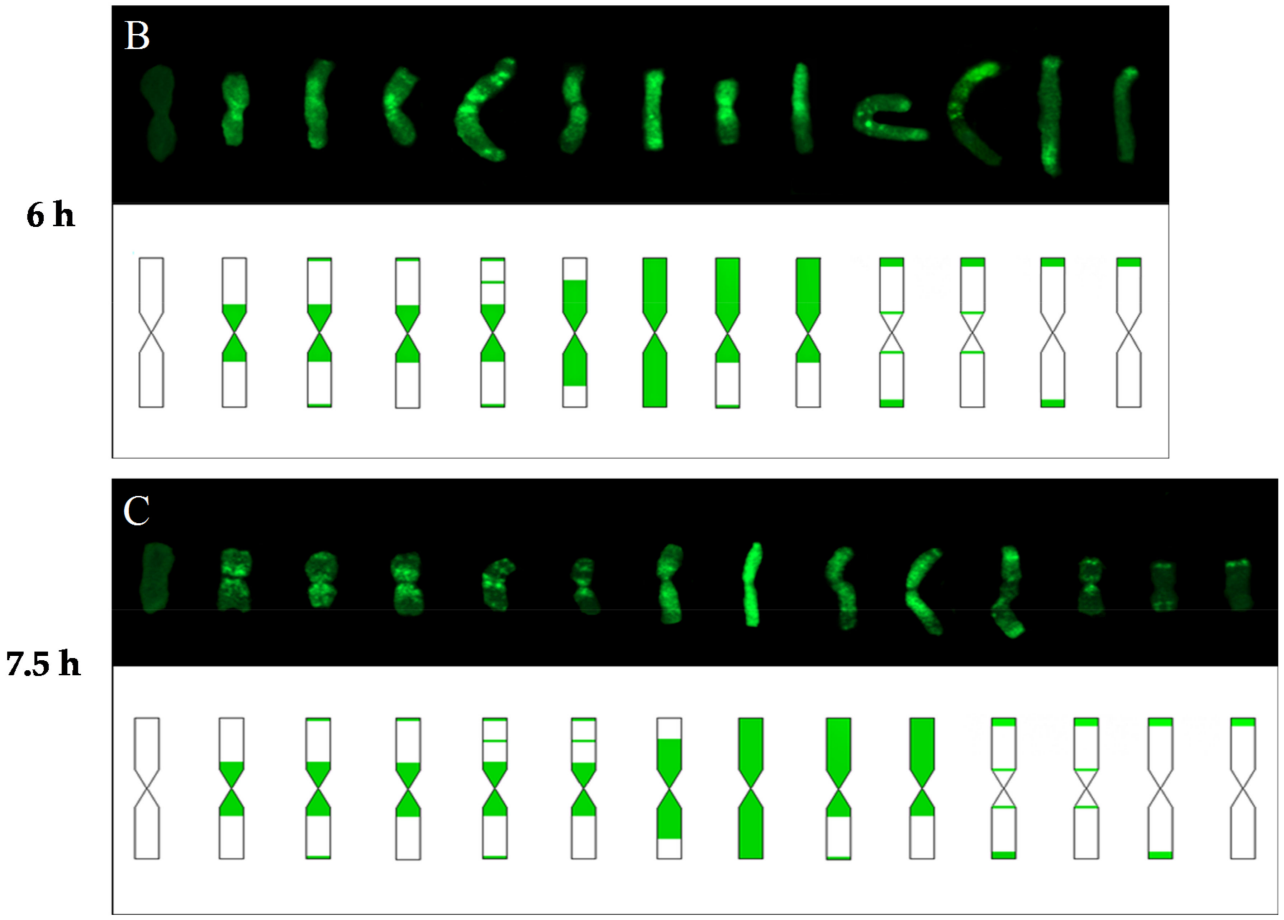
Types of replication patterns in the barley chromosomes in the γ ray-treated roots at 4 h (**A**), 6 h (**B**), and 7.5 h (**C**) after EdU incorporation. Chromosomes with different replication patterns and their corresponding schematic presentation are shown. Only one morphological type of chromosome is presented in order to simplify the scheme.

The differences in the chromosome replication pattern for a particular experimental group and at specific time points were observed. Figure 4, Figure 5 and Figure 6 showed the replication patterns in the chromosomes in the control, γ ray-, and MH-treated roots at specific time points: 0, 2, 4, 6, 7.5, and 10.5 h. Chromosomes with different replication patterns and their corresponding schematic presentation are shown. Only one morphological type of chromosome is presented in order to simplify the schemes.

In the control at 4 h, large replication signals were observed in the chromosome centromeric regions and small signals were also observed in the distal and interstitial regions (Figure 4). These chromosome regions are replicated in the late S-phase, passed the G2-phase as a first, and thus can be observed in the first labeled metaphases. At 6 h, five more chromosome replication patterns were observed than at 4 h. Chromosomes that were completely labeled were observed most often. Another pattern was characterized by replication signals that covered one chromosome arm and the pericentromeric region of the second arm, sometimes with additional distal signal(s). Chromosomes that had small replication signals in the distal and centromeric regions were also observed. Only two new replication patterns were observed at 7.5 h—both with signals in the distal regions, which is characteristic for early S-phase cells. In addition to the new types of signals, the most-frequent replication pattern in this hour was also fully labeled chromosomes. The chromosome labeling that was characteristic at 6 and 7.5 h involved the regions that had been replicated in the early, middle, and late S-phase. Generally, at 10.5 h, the same chromosome replication patterns were observed as at 7.5 h. The most frequently appearing pattern of replication at this time was characterised by the signals in the distal parts. However, at 10.5 h, new chromosome replication patterns were additionally observed—with only one sister chromatid labeled within an individual chromosome. The other types of patterns that were observed at 10.5 h were chromosomes with both sister chromatids labeled, although each of them was labeled in a different part.

After treatment with maleic hydrazide, only six replication patterns were observed. Figure 5 shows the different replication patterns in the MH-treated root at 10.5 h. No replication signals were observed at the earlier time points. Fully labeled chromosomes were the most frequently observed.

The same thirteen replication patterns were observed in the γ ray-treated roots as in the control cells (Figure 6). Basically, the same patterns of replication dominated at given hours as in the control cells. However, differences in their occurrence at specific time points in the control and γ ray-treated roots were observed. Interestingly, the labeling of whole chromosomes in the control was observed at 6 h, whereas in the γ ray-treated roots, it was already observed at 4 h. Similarly, the chromosomes with labeling in the terminal regions of a chromosome were observed at 6 h in the γ ray-treated roots, whereas in the control, they were observed at 7.5 h. Additionally, the chromosomes with Alexa Fluor 488 fluorescence in large bands in the pericentromeric regions were observed at 4 h and 6 h in the control and at 4, 6, and 7.5 h in the γ ray-treated roots.

**Figure 7:**
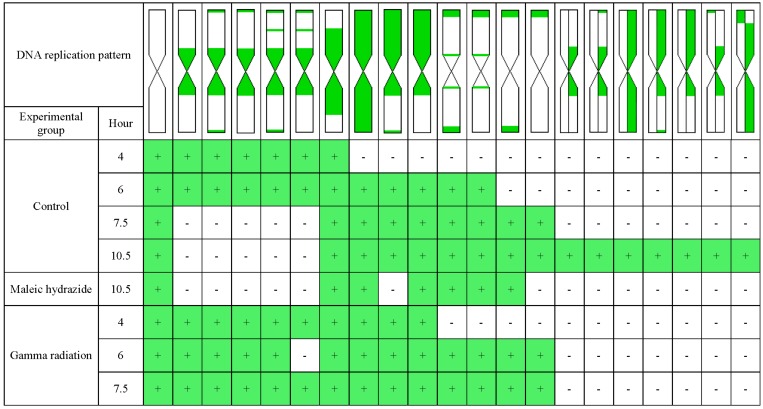
Comparison of the replication patterns in the barley chromosomes in the control, and MH- and γ ray-treated roots, and their occurrence at different time points after EdU incorporation. “+” means the presence of particular DNA replication pattern, “−” means the lack of particular DNA replication pattern.

In the present study, the application of 5-ethynyl-2′-deoxyuridine (EdU) incorporation and detection allowed differences in the replication pattern at different time points after EdU incorporation in all of the experimental groups to be determined. Figure 7 presents a comparison of the replication patterns in the barley chromosomes in the control, and MH- and γ ray-treated roots, at different time points after EdU incorporation.

## 3. Discussion

In present study we analyzed the distribution of a DNA replication pattern on the *H. vulgare* ‘Start’ variety chromosomes, as well as its relationship to the DNA damage, using EdU method. Different replication patterns were observed in the chromosomes in the barley roots within individual metaphases. This may be due to differences in the DNA packing of individual chromosomes. It is well known that euchromatin and heterochromatin regions replicate at different times during the S-phase. Individual barley chromosomes are characterized by a different localisation of constitutive heterochromatin, as was demonstrated by the Giemsa C-banding technique [9]. Therefore, the chromosomes belonging to one metaphase plate may have a different DNA replication pattern. Due to results, we can state that DNA replication in barley chromosomes begins in the terminal chromosome regions (early S-phase), after which its pattern is observed in whole chromosomes, and at the end—in the centromeric regions (late S-phase). These analyses indirectly provide information about the localisation of euchromatin and heterochromatin in the chromosomes of the *H. vulgare* ‘Start’ variety. Since it is known that DNA replication starts in euchromatin and then continues in the heterochromatin regions, this implies the presence of transcriptionally active genes in the terminal chromosome regions and the inactive heterochromatin in the centromeric regions. A similar localisation of euchromatin and heterochromatin has previously been shown using the BrdU incorporation and detection methods, and fluorescence, in situ hybridisation with centromeric and telomeric probes [10].

Thirteen types of replication patterns were distinguished in the control barley chromosomes with EdU incorporation and detection, whereas only five patterns had previously been observed using BrdU incorporation and detection [11]. This is probably due to the possibility of discovering small signals with EdU method by using a small size detection azide and eliminating the denaturation step, which is necessary for detection of BrdU. For example, the chromosomes with just centromeric signals were observed using BrdU, whereas patterns with centromeric signals, as well as terminal, or terminal and interstitial, signals, were observed using the EdU method. The accuracy of this method allowed one to notice the differences between the occurrence times of individual replication patterns in control and treated cells. This can prove that EdU can potentially be applied to studying the effects of mutagens on cell cycle disturbances, especially in plant species that are characterized by small chromosomes.

Beside the typical replication signals comprising two chromatids, specific new chromosome replication patterns were observed in control at 10.5 h—with only one sister chromatid labeled within an individual chromosome. This labeling pattern was characteristic for the cells in which the DNA replication occurred twice—first, the DNA synthesis in the presence of EdU, and the second, without EdU. Other new patterns observed at this time were chromosomes with both chromatids labeled, although each of them in different localisation. This is characteristic of the sister chromatid exchange (SCE), which is commonly known in plants, as well as in animals and humans. The occurrence of SCEs was first demonstrated using autoradiography, and later a procedure that had a much greater resolution using BrdU was introduced [12]. It is known that BrdU itself induces the SCEs due to its mutagenic effect. Until now, there is no data on the possible application of EdU in the SCE method and, consequently, on its effects on the induction of sister chromatid exchanges.

Additionally to the main aim, the results of this study provided new data about the duration of the G2-phase and the cell cycle of the *H. vulgare* ‘Start’ variety cells. The first labeled metaphases in the control roots were observed at 4 h after the incorporation of EdU, while at 2 h after the incorporation of EdU no labeling has been observed within the chromosomes. This indicates that the duration of the G2-phase is between 2 h and 4 h. The replication pattern with labeling in only one chromatid of the chromosome was observed at 10.5 h. This proves that during 10.5 h, in addition to the G2-phase, there was also a complete next cell cycle without the presence of thymidine analogs. Considering that the length of the G2 phase is more than 2 h, it could be concluded that the duration of the cell cycle in *Hordeum vulgare* ‘Start’ variety is at most 8.5 h. It should also be emphasized that the pattern of replication with labeling in only one chromatid within the chromosome could also be observed in the earlier hours after incorporation of EdU—between 7.5 h and 10.5 h. It is also possible that the duration of the G2 phase may be longer than 2 h. In both cases, this would mean that the duration of the cell cycle in this variety of barley can be even shorter than 8.5 h. The experiments for the ‘Start’ variety were planned according to the mean duration of the cell cycle of other varieties of barley, e.g., ‘Brage’—10.4 h [13], ‘Sultan’—12.4 h, ‘Maris Otter’—12 h [14], and ‘Amethyst’—9.2 h [15]. The results of this study indirectly prove that the duration of the cell cycle of the *H. vulgare* ‘Start’ variety is definitely shorter than in the previously described varieties of this species.

The analyses showed the differences in the chromosome replication pattern for a particular experimental group and at specific time points. After treatment with maleic hydrazide, only six replication patterns were observed. First chromosomes with replication patterns in the MH-treated roots were observed only at 10.5 h. This may be due to the mechanism of MH action, namely, its influence on the synthesis of the nucleic acids and enzymes that are involved in the mitotic spindle [16,17]. The mitotic activity can even be totally stopped after MH treatment [18]. The patterns of replication that were observed after MH treatment were characteristic of the regions that have been replicated in the early and middle S-phase, even though the patterns for the late S-phase should be observed first. This may indicate that MH led to a complete cell cycle arrest in the cells that were in the late S-phase during the incorporation of EdU. After treatment with MH, the observed patterns differed considerably from patterns that were observed at the same time point in the control cells. No replication patterns with signals involving one sister chromatid have been observed, thus excluding a second DNA synthesis without the presence of EdU. MH is a clastogenic and mutagenic agent that may cause the S-phase to be extended. We found that the first labeled metaphases in the MH-treated cells were observed at 10.5 h while in the control cells already at 4 h. This may be due to both the extension of the S-phase or a delayed G2/M transition. For the first time, comparing the time of appearance of the first labeled metaphases in control and treated material, we can precisely evaluate that after MH treatment the duration of cell transitions from the S-phase of the cell cycle to mitosis was extended for about 6.5 h.

Differences at specific time points in the control and γ ray-treated roots were also observed. The labeling of whole chromosomes in the control was observed at 6 h, whereas in the γ ray-treated roots, it was already observed at 4 h. Similarly, the chromosomes with labeling in the terminal regions of a chromosome were observed at 6 h in the γ ray-treated roots, whereas in the control, they were observed at 7.5 h. It is known that γ ray acts during the G1, S, and G2 cell cycle phases. We conclude that γ ray can lead to a shortening of the S-phase or acceleration of the G2/M transition, which is judged by the presence of the replication patterns that are characteristic for the early and middle S-phase earlier than in the control cells. This physical mutagen may also have the opposite effect—extending the S-phase of the cell cycle or G2/M transition, as was evidenced by the presence of replication patterns that are characteristic for the late S-phase longer after the end of the EdU incorporation than in the control cells.

Summarizing, differences in the temporal distribution of the replication patterns between the control, γ ray-, and MH-treated roots were found. Slight differences were observed regarding the replication patterns in the control and γ ray-treated roots, while differences in replication patterns in the control and MH-treated cells were more significant. The results obtained in this work are consistent with those previously obtained by Kwasniewska et al. [2] during an analysis of the replication process in the barley nuclei. MH has a stronger effect on DNA replication than γ radiation. It was demonstrated that treatment with MH and γ ray did not change the characteristic S-phase patterns in the nuclei; however, the frequencies of the S-phase labeled cells after mutagenic treatment were different than in the control cells. The results of this study on the pattern of replication in barley chromosomes also confirm that no new replication patterns are observed after mutagenic treatment. However, differences were found in the temporal distribution of the replication patterns between the control, γ ray-, and MH-treated roots, as well as differences in the frequency of the labeled metaphases. Moreover, previous studies on the replication patterns in barley cells have also shown that the frequencies of EdU-labeled cell nuclei in the early, middle, and late S-phase were different in the control cells and cells that had been treated with mutagens. After treatment with MH, a significant increase has been observed in the frequency of labeled cells in the middle S-phase. This may indicate an extension of the S-phase of the cell cycle after treatment with this chemical mutagen, as is also confirmed by the data presented in this paper. After treatment with γ ray, in turn, a significant increase in the frequency of labeled cells in the late S-phase has been observed before, which confirms that γ ray can extend the late S-phase in barley cells. This is followed by the observation that the pattern of replication in chromosomes that are characteristic for late S-phase phase occurs longer after the incorporation of EdU than in the control cells.

## 4. Materials and Methods

### 4.1. Mutagenic Treatment

Seeds of the barley (*Hordeum vulgare,* 2*n* = 14) ‘Start’ variety were used as the plant material. Maleic acid hydrazide dissolved in water (4 mM MH; Sigma-Aldrich, St. Louis, MO, USA, CAS 123-3301) and a gamma ray (175 Gy) were used for the mutagenic treatment. The mutagen doses used in the study had been applied in previous experiments in which their cytogenetic effects were well characterized [7,8]. Before chemical treatment, the barley seeds were pre-soaked in distilled water for 8 h and then treated with MH for 3 h. Two treatment experiments using MH were performed. After the treatment, the seeds were washed three times in distilled water and then germinated in Petri dishes lined with moist filter paper at 21 °C in the dark for 3 days. Our previous findings showed that the cytogenetic effect of MH treatment is observed in the roots of three-day seedlings [7]. The irradiation was performed at the International Atomic Energy Agency, Seibersdorf Laboratory, Austria in 2015. For about half a year, the seeds had been kept in a refrigerator. Keeping the seeds in the 4 °C is commonly practiced (also in mutagenesis studies). Additionally we ourselves confirmed, in the number of cytological analyses, that during this storage the cytogenetic effect of irradiation still persisted and did not diminish. We observed numerous breaks in the chromosomes and the formation of dicentric chromosomes, which is one of the characteristic effects of the mutagenic effect of gamma radiation (these results are not presented here). Similar storage time has been used in our previous experiments [8]. After irradiation, the seeds were pre-soaked in distilled water for 8 h and germinated in Petri dishes at 21 °C in the dark. Two experiments were performed for each mutagenic treatment. 

### 4.2. EdU Incorporation and Detection

The incorporation and detection of 5-ethynyl-2′-deoxyuridine (EdU; Click-iT EdU Imaging Kits Alexa Fluor 488, Invitrogen, Carlsbad, CA, USA) were applied according to the manufacturer’s procedure with minor modifications. The 3-day barley seedlings were incubated for 30 min in the dark in a 10 mM EdU solution. The seedlings were rinsed in distilled water 2 × 5 min and fixed in ethanol:glacial acetic acid (3:1) for 2 h at room temperature (RT) and 2 h at 4 °C at 0, 2, 4, 6, and 7.5 h after EdU incorporation. Additional fixation time points—9 h and 10.5 h—were applied for the control and MH-treated roots. The times for fixation and examination of replication pattern after the end of EdU incorporation were selected according Kakeda and Yamagata [11] with modifications. The roots of the seedlings were used as the source of the meristems for the investigations. For chromosome preparation, the material was washed with a 0.01 mM sodium citrate buffer (pH 4.8) for 30 min and digested with 2% cellulase (*w*/*v*, Onozuka, Serva, Heidelberg, Germany) and 20% pectinase (*v*/*v*, Sigma-Aldrich) for 2 h at 37 °C. After digestion, the material was washed with a sodium citrate buffer for 30 min. Squash preparations were made in a drop of 45% acetic acid. After freezing and removing the coverslips, the slides were dried. Prior to EdU detection, the slides were permeabilized with 0.5% Triton X-100 for 20 min and then washed in PBS at RT. The slides were incubated for 30 min at RT in an EdU reaction cocktail (Click-iT EdU Imaging Kits Alexa Fluor 488, Invitrogen), which was prepared according the manufacturer’s procedure. For one sample reaction, the following components were added: 43 µL of a 1× Click-iT reaction buffer, 2 µL of CuSO_4_ (Component E, 100 mM), 0.12 µL Alexa Fluor 488 azide (Component B), and a 5 µL reaction buffer additive (Component F). After 2 × 5 min washes, the slides were stained with 2 µg/mL DAPI (Sigma-Aldrich), washed with PBS, and mounted in a Vectashield medium (Vector, Burlingame, CA, USA).

### 4.3. Analysis

Preparations were examined using a Zeiss Axio Imager.Z.2 wide-field fluorescence microscope (Zeiss, Oberkochen, Germany) equipped with an AxioCam Mrm monochromatic camera (Zeiss, Oberkochen, Germany). For the analyses of the distribution of the EdU pattern on the barley chromosomes, images were captured and processed using Adobe Photoshop 4.0 (San Jose, CA, USA). The frequencies of the metaphases with Alexa Fluor 488 signals were calculated. For each experimental group (control, MH, and γ ray) and individual time point, 100 metaphases were evaluated. The number of analyzed plants was different depending on the experimental group. Due to significant decrease in the mitotic index in roots after MH treatment, about 10 plants have been used for each hour after the incorporation of EdU. For control and gamma-irradiated samples, not less than 5 plants were evaluated.

The significance of differences between control and treated groups was evaluated with Student’s *t*-test (*p* < 0.05).

## 5. Conclusions

This work demonstrates the usefulness of the EdU method in a detailed analysis of the replication patterns in barley. Such a high resolution of the EdU method indicates that it can be used for more precise analyses of cell cycle disturbances, not only for plant species with large chromosomes, but especially for those with small chromosomes. Analyses of the distribution of the signals of DNA replication on the chromosomes revealed relationships between DNA replication, the chromatin structure, and DNA damage, and thus they are more informative than studies using non-dividing cells. We proved that MH has a stronger impact on replication than γ ray by its action of extending the duration of cell transitions from the S-phase of the cell cycle to mitosis by 6.5 h. Data regarding the duration of the cell cycle in the *H. vulgare* ‘Start’ variety are also presented for first time.
